# The need for sharps boxes to be offered in the hospital setting for people who use substances: Removing sharps boxes puts all of us at risk

**DOI:** 10.3389/frhs.2023.1113163

**Published:** 2023-04-06

**Authors:** Cheryl Forchuk, Michael Silverman, Abraham Rudnick, Jonathan Serrato, Brenna Schmitt, Leanne Scott

**Affiliations:** ^1^ Mental Health Nursing Research Alliance, Lawson Health Research Institute, London, ON, Canada; ^2^ Arthur Labatt Family School of Nursing, Western University, London, ON, Canada; ^3^ Schulich School of Medicine & Dentistry, Western University, London, ON, Canada; ^4^ London Health Sciences Centre, Victoria Hospital, London, ON, Canada; ^5^ St. Joseph’s Health Care, Victoria Hospital, London, ON, Canada; ^6^ Department of Psychiatry, Dalhousie University, Halifax, NS, Canada

**Keywords:** harm reduction, sharps boxes, hospitals, substance use, nursing, safety, nurse education, sharps containers

## Abstract

**Introduction:**

Substance use can occur in the hospital setting among people with substance use disorder, including intravenous use. However, the provision of sharps boxes is not typically offered in Canadian hospitals. This study set out to explore the current issues due to the lack of harm reduction in the hospital setting.

**Method:**

Thirty-one health care professionals participated in virtual one-to-one interviews and focus groups regarding harm reduction in hospital. The issue of sharps box removal was highlighted as a concern. A secondary ethnographic thematic analysis explored this theme in more detail. A scoping review of the literature observed additional considerations.

**Findings:**

Sharps box removal was commonplace for people who were known to be, or suspected of, using substances. Sharps boxes only to be used for medical purposes and fears of box tampering were cited as reasons for removal. Health care professionals noted that patients would have to use sharps boxes situated elsewhere. The scoping review revealed that needlestick injuries for hospital staff decreased with greater access to sharps boxes in hospital. Injuries can be addressed through safer disposal practices. Modern designs of sharps boxes and educational initiatives have been found to be successful in sharps disposal compliance and reductions in related injuries.

**Discussion:**

Ensuring equitable access to sharps boxes would help to reduce unsafe needle discarding which can lead to needlestick injuries for hospital staff and potentially other patients. Education would be a key step in furthering understandings of the importance of sharps boxes and harm reduction as a whole.

## Introduction

Harm reduction strategies (including counseling, opiate substitution therapy, safe supply, supervised consumption, among other interventions), are currently lacking in many hospital settings in Ontario, and in North America more broadly. This gap directly impacts those with substance use disorder who are admitted to hospital, including those who use methamphetamine. This includes psychiatric hospitalizations as well as medical ward admissions. Patients have reported to continue using substances within the hospital setting to avoid negative circumstances such as inadequate pain management, withdrawal, loneliness, sadness and boredom (1, 2). Of particular importance is the lack of sharps boxes (also referred to as sharps containers or sharps bins) in many of the hospital rooms of patients with substance use disorder. This is problematic for a number of reasons. Notably, individuals who inject substances in hospital have nowhere to dispose of their used equipment. They are often forced to discard their needles in bedsheets and linens, through regular waste disposal mechanisms such as garbage bins, or transport used needles to another location where a sharps box may be situated. This phenomenon can place the patients themselves, other patients, health care workers, and cleaning staff at heightened risk of needlestick injuries (NSI).

This paper will first outline findings regarding sharps box removal from a study being conducted into implementing harm reduction strategies and philosophies in the hospital setting. This will then be followed by a review of the literature concerning the dangers of NSI including prevalence and health consequences, and the design of sharps boxes to allay fears of tampering. This paper then explains why there is a need to prevent the removal of sharps boxes and the implications of such, including education for hospital staff.

## Findings of the methamphetamine harm reduction study

As part of a 4-year study into harm reduction strategies in hospital settings for methamphetamine use, health care professionals participated in focus groups and individual interviews to discuss how strategies would be implemented. This included staff from both psychiatric and medical ward settings. Current issues when caring for people who use methamphetamine were also discussed. It was reported by multiple health care professionals that they had been instructed to remove sharp boxes from the rooms of patients known or suspected to use substances.

*“We remove sharps containers from within the room. When we have patients who inject drugs, which I think actually leads to an increased likelihood of, like, needle incidents like sharp incidents for staff and obviously they can't dispose of their sharps as well.”*

*“But don’t we take, we take out the bins, don’t we?”*

*“Yeah. Oh yeah. Yeah we take out the bins and they, they get plastic cutlery. If they have a known recent history of substances.”*

*“…We treat them so different.”*

It was stated that sharps boxes were for medical use only and not for any other needle discarding purpose.

*“There's nothing like dedicated to that right now. Like the sharps containers are mostly, I think for, like, supposed to be for medical use. It's not necessarily this, this one's for substance needles right.”*

Concerns as to what might occur if the sharps boxes were left in the rooms included theft and drug-seeking behaviour of potentially discarded vials.

*“The concern is they would try to go into the sharps box and take maybe the vials of hydromorphone, for example, out of the box.”*

As a result, sharps boxes are not easily accessible to people who use substances. This would mean that patients would have to move around the hospital and search for a bin should they wish to discard safely.

*“Yeah. There are bins in the bathrooms, the public bathrooms, but that would be… They’d have to carry it to the public bathroom or go into another patient’s room to access a bin.”*

This was also done to comply with hospital policies of abstinence from substance use while admitted. In some discussions with health care professionals and at Advisory Group meetings, removing sharps boxes was thought to have been a form of harm reduction. But this only increases the risk of needles being unsafely discarded thereby putting other patients, health care professionals, housekeeping staff, and even the patient themselves at risk of injury. For example, members of staff from the linens and housekeeping department have reported large amounts of sharps found, including needles. Upon reporting the finding of sharps box removal to the study's Advisory Group, it was noted that removal of sharps boxes was conducted for staff safety in case the boxes were used as weapons or projectiles. However, an internal analysis of hospital incident reports involving sharps boxes in the previous 5 years did not yield any instances of boxes being used as projectiles or weapons.

In light of these findings, a resolution was submitted to the Registered Nurses Association of Ontario to recommend the non-removal of sharps boxes. The resolution was passed with an approved vote of 86%. Within the institution of the research team, an infographic explaining and promoting the non-removal of sharps boxes was commissioned for distribution across all wards. An article explaining why this practice needs to change and why senior management is wanting the non-removal of sharps boxes has also been prepared for a hospital-wide newsletter via the hospital's media relations department. Further education for health care professionals on substance use disorder, harm reduction, and withdrawal, has also been approved. These new educational sessions have been designed to provide staff with additional knowledge and resources in caring for people who use substances. The sessions are also co-coordinated with lived experience educators who provide peer support at local safe consumption sites and will present their personal experiences for successful care within the hospital setting.

A scoping review of the literature was also conducted to explore current understandings and guidance in using sharps boxes. Five databases were utilized for this literature search including Google Scholar, JSTOR, ProQuest, PubMed and Scopus. Reference lists of identified articles were also used for further searches. Key search terms included “sharps box”, “sharps deposit box”, “needle deposit box”, “needle deposit box” and “hospital”. Other search terms for specific areas of interest included “removal”, “sharps safety”, “needle safety”, “safe needle disposal”, “sharps injury”, “needlestick injury”, “use or uptake”, “report” and “risk”. The inclusion criteria for articles were as follows: (1) had to be in a hospital or clinic setting, or in the community, (2) the population under study had to be hospital or clinic staff, patients, or people who use substances, (3) peer reviewed and (4) English language only. No exclusion criteria were set for the study design, geographical location of the study, or year of publication.

## Dangers of needlestick injuries

A number of studies have looked at prevalence and context of sharps and NSI in various hospital settings. A recent metanalysis of worldwide data revealed that lifetime NSI prevalence among health care workers was 56%, and 32% for the previous year (3). The authors note that socioeconomic factors may play a role as significantly higher rates of NSI were observed in developing countries. A review of eight studies involving over 7,000 healthcare workers found that sharps injury rates were highest in nurse and doctor cohorts (4), but the data is mixed as to which type of health care professions experiences the most NSI. Bhargava et al. (5) reported that although the overall NSI rate for all staff was 44%, which is still alarmingly high, 58% of doctors reported an NSI compared to 36% of nurses. However, a review which analyzed data from the Exposure Prevention Information Network found that 56.8% of all sharps injuries were reported by nurses, while 26.8% of injuries were reported by physicians (6). Highest NSI prevalence in nursing staff has also been reflected in other studies (7, 8). Nursing students have been found to experience NSI with one meta-analysis revealing an incidence of 35% among nursing students across 32 studies (9).

Cheung et al. (10) conducted a cross-sectional survey study amongst nursing students in Hong Kong, and found that NSI occurred most often in medical or surgical wards. In the past, McCormick et al. (11) obtained data regarding reported sharps injuries from a hospital following the introduction of wall-mounted sharps containers, and found that sharps injuries were most common in nursing personnel (58%), and in inpatient units (71%). In this study, collection and disposal of waste linen and procedure trays were the activities most often associated with NSI (11). Importantly, in this study, making disposal units available at every bedside reduced injuries from needle disposal two-fold.

Risk of NSI in relation to various factors has been extensively studied, and a number of arguments have been made in support of the provision of sharps boxes in patient rooms. NSI have been found to be responsible for nearly 40% of Hepatitis B and C infections among health care workers worldwide (12). Policy change geared towards safe disposal practices (including the provision of sharps boxes in patient rooms) is associated with decreased risk of injury. After the introduction of a series of safe disposal recommendations and regulations in a number of U.S. hospitals in 1987, Perry et al. (13) examined changes in disposal-related injury patterns and found a 40% decline in percutaneous injuries in patient rooms between 1993 and 2007. Researchers also noted a 61% decline in rates of housekeeper injury per 100 occupied beds between the 2 data collection points (13).

Immediate disposal resulting from having sharps boxes in close proximity to the point of use has been associated with a decreased rate of NSI. Cheung et al. (10) found that immediate needle disposal was significantly associated with decreased odds of 12-month prevalence of injury. Close proximity of containers also increases convenience and speed of disposal, both of which decrease the length of exposure to used sharps (14). Size of container has also been found to impact risk—Richard et al. (15) found that smaller containers were less likely to be overfilled. In terms of environmental impact, proper disposal can have positive environmental implications by decreasing the number of needles and other substance use equipment that are sent to the landfill (14).

## Design of sharps boxes

Reduced risk of injury has also been associated with changes in sharps box design, including larger openings, incorporating passive overfill protection, and hand-entry prevention. Container-associated sharps injuries fell from 11.4% to 2.2% over 2 years (2006–2008) following the replacement of single-use sharps boxes with advanced engineered ones in 14 hospitals (16). Grimmond et al. (17) also noted a 32.7% reduction in total sharps injury rates per 100 occupied beds following the implementation of a Sharpsmart container program (with the previously mentioned features) and accompanying sharps injury education for staff in various Australian and UK hospitals. The proportion of sharps injuries that were container-related also fell from 11.6% pre-implementation to 1.1% post-implementation (17). Grimmond and Naisoro (18) conducted a 6-year three-phase transition from disposable sharps containers to a wall-mounted, advanced engineering container in patient rooms. Compared to phase 1 (disposable containers), data collected in phase three (wall-mounted containers) saw an 83.1% statistically significant decrease in disposal-related sharps injuries.

## Why sharps boxes provision is needed

Although most of the literature on sharps box provision is focused on safety for health care staff, the implications for patients are equally as important. Sharps box provision is a critical component of harm reduction initiatives, alongside other strategies like counseling, safe supply, peer support, needle exchanges and education/training. A syringe services program which incorporated counseling, safe supply, and proper disposal in community sharps boxes noted an increase in safely disposed needles, as well as a decrease in unsafe substance use behaviours, such as needle sharing (19). The Canadian Research Initiative on Substance Misuse (CRISM) also stressed the importance of accompanying harm reduction education initiatives with the provision of readily accessible sharps containers and personal sharps containers provided to patients directly, as well as instruction on proper disposal methods (20). Indeed, people tend to use sharps boxes when they’re available. The provision of sharps boxes in the community has been associated with fewer improperly disposed sharps in multiple studies (21, 22). Less substance use equipment discarded unsafely has subsequently been related to a sense of a cleaner community (19).

Use and uptake of sharps boxes in the community is influenced by public opinion and acceptance, particularly in areas where people who use substances may use more commonly. Devaney and Berands (23) conducted an evaluation of a number of sharps boxes within the bathrooms of eleven businesses in Melbourne, Australia. Many interviewees reported that the containers had reduced or stopped inappropriate disposal of needles in public bathrooms. Nine out of the 11 businesses chose to take up the cost of servicing the disposal containers post-study. By normalizing the use of sharps boxes in the community, public support and understanding of the benefits of sharps boxes may also help to facilitate the concept of sharps box provision to patients.

Despite the extensive body of literature looking at safety, several issues have also been identified in the literature in relation to the provision of sharps boxes in patient rooms. Several studies have noted under-reporting of sharps injuries as a barrier to fully understanding the burden of NSI (4). Other studies have reported increases in NSI reporting after greater awareness and enhanced training. Richard et al. (15) observed an increase in total injuries reported after introducing sharps containers and staff training, although the proportion of injuries related to improper disposal was lower overall. Another issue that has been discussed is the importance of accompanying sharps box introduction to staff and patient education on proper use in order to minimize issues related to improper disposal using newly installed sharps containers (20).

Concerns related to theft of containers and their contents have also been raised in the literature, albeit rarely and not necessarily in relation to patients who use substances. Gwyther (24) discussed the need for fixing containers to walls due to a concern for container theft and sale of contents. Additionally, Grissinger (25) emphasized the need for small openings on containers and consistent surveillance in areas that have sharps boxes in order to reduce the likelihood of drug diversion by staff or patients. Concerns regarding the use of a sharps box as a projectile or weapon have also been mooted but there is no literature on this and this research team found no evidence based on local incident reports since 2017. Regardless, many of these concerns are founded in outdated and inaccurate information about the ways in which sharps boxes work and the nature of substance use as a mental illness, emphasizing the need for more widespread education and training initiatives for those who use and dispose of sharps.

## Education for health care staff

Staff education and training initiatives have been identified as critical components to the successful operation of a sharps box program. Emanating from the qualitative findings of the Methamphetamine Harm Reduction project was the practice of removing sharps boxes from the rooms of patients who were known or suspected of using substances. Enhanced education regarding harm reduction and what it entails was recommended by health care providers. Providing reassurance that modern sharps boxes do not allow for needles to be retrieved easily would also be beneficial. Other studies have revealed that enhanced education regarding the disposal of sharps can also have a significant effect on staff safety. Bijani et al. (26) revealed a significant reduction in the number of NSIs and exposure to blood and bodily fluids among nurses who received a continuing education program but no change was noted for the control group. Hussain et al. (27) reported an increase in compliance with safe disposal practices from 44% in 2019 to 82% in 2020 following a series of meetings with staff members regarding proper sharps disposal. Other studies have also documented the importance of a culture of sharps safety and disposal. In interviews with staff members conducted by Judge et al. (28) some interviewees stated that safe use of disposal devices was compromised by poor or absent sharps disposal behaviour. Similarly, Kable et al. (29) found that nurses felt more inclined to dispose of sharps safely when others were performing sharps disposal behaviours and when they felt supported to do so through their organization.

Education can help to reduce the number of NSI as a result of better-informed practices. In one study, NSI significantly reduced by more than half among trained health care workers who demonstrated a statistically significant lower relative risk ratio of 0.06 after receiving an awareness education program on biohazard risks (30). Additional training and education can not only improve practices but also support knowledge which could then influence good practice. Training could also support skill acquisition which can enhance health care workers’ proficiency in completing tasks safely. Chen et al. (31) reported that physicians with high self-efficacy engaged in NSI prevention behaviours at times of both high and low job demand. It would be worth considering for future policy decision-makers and organizational leadership to consider additional education ventures to ensure a culture of NSI awareness and for hospital staff to be skilled in NSI prevention.

## Discussion

As a result of removing sharps boxes from patient rooms, people who use methamphetamine may feel a sense of mistrust from their health care provider. Feelings of mutual mistrust were reported in the current study. These feelings could then result in perceptions of stigma or discrimination which would negatively affect the therapeutic relationship. Stigma has been found to result in an aversion to care, prompt people to discharge themselves against medical advice, and deter people from disclosing substance use to their care providers (32–34). The provision of a sharps box also provides the individual with a level of autonomy which can help to engage them in their care. This granting of autonomy could also provide a greater sense of acceptance and trust between the patient and the health care system.

An additional implication could be to go beyond the non-removal of sharps boxes and explore the possibility of gifting personal sharps boxes. Miskovic et al. (35) reported that personal biohazard bins (along with injection and smoking kits) were successfully provided 24 h a day to patients at a small hospital specializing in HIV treatment in Toronto, Ontario, Canada. Brooks et al. (36) also successfully provided harm reduction kits including personal sharps boxes in a hospital on Edmonton, Alberta, Canada. In both studies however, no evaluation was conducted on the containers themselves. Future research is needed to assess the effectiveness and efficacy of safety in the provision of personal sharps boxes. In the broader scheme of harm reduction, services in the hospital setting have been established in various cities in Canada including Vancouver (37, 38), Edmonton (39), and Toronto (40). Strategies such as harm reduction kits and needle exchange programs have also been implemented on hospital grounds in other countries such as the United States (41, 42), Australia (43), and Scotland (44). These studies indicate that harm reduction strategies can feasibly be adopted into the hospital setting. Future harm reduction services should consider and include the provision of personal sharps boxes to improve safety for people who use substances.

Ideally a personal sharps box would be provided on discharge to people who use substances and of a convenient size for ease of transportation. Training and instructions on how to dispose of sharps safely into the box would also be recommended. Should the box become full while in hospital, staff would need to assist the individual in disposing of it and replacing it with a new, empty box. It would be preferable for people to keep the sharps box throughout their admission and post-discharge in order to maintain safety in the community. Information would also need to be provided on what to do with the box once full after discharge such as taking the box to a pharmacy. Community resources would need to be made available to empty and sterilize, or replace, sharps boxes. Future strategies and interventions would need to ensure that continuity of care and safety is established between the hospital setting and support services in the community.

## Conclusion

The literature indicates that providing people who use substances with the option of a sharps box would increase patient as well as staff safety. It is therefore highly encouraged that hospital leadership revise the current culture that encourages the removal of sharps boxes from the rooms of people with substance use disorder to maintain abstinence. The maintenance of abstinence whilst admitted, represents an unrealistic expectation for this patient population, especially substances such as methamphetamine for which substitution therapy is not generally available. Education to reduce stigma and bias would be an essential first step in furthering understanding of harm reduction. Modern sharps boxes are reliable and effective tools in preventing unsafe discarding and making them accessible allows for a much safer environment to all in the hospital setting. Ensuring access to sharps boxes would help to prevent the unsafe discarding of needles which puts health care providers, personal support workers and linens staff at risk of NSI as well as patients.

## Data Availability

The datasets presented in this article are not readily available because we do not have permission from our Funder or our Ethics Board to share our data freely. Our agreements with both institutions state that data can only be shared with co-investigators/partners for analysis purposes. This was also stated in our Consent forms with participants. We would be open to collaborating on future papers with other research teams where we could share data as partners and add them to our agreements with the Funder and Ethics Board. Requests to access the datasets should be directed to Jonathan Serrato, jonathan.serrato@lhsc.on.ca.
